# Typing *Clostridium difficile *strains based on tandem repeat sequences

**DOI:** 10.1186/1471-2180-9-6

**Published:** 2009-01-08

**Authors:** N Henning Zaiß, Maja Rupnik, Ed J Kuijper, Celine Harmanus, Dolf Michielsen, Koen Janssens, Ulrich Nübel

**Affiliations:** 1 Robert Koch Institute, Wernigerode, Germany; 2 University of Maribor, Maribor, Slovenia; 3 Institute of Public Health Maribor, Maribor, Slovenia; 4 Department of Medical Microbiology, Center of Infectious Diseases, Leiden University Medical Center, Leiden, The Netherlands; 5 Applied Maths NV, Sint-Martens-Latem, Belgium

## Abstract

**Background:**

Genotyping of epidemic *Clostridium difficile *strains is necessary to track their emergence and spread. Portability of genotyping data is desirable to facilitate inter-laboratory comparisons and epidemiological studies.

**Results:**

This report presents results from a systematic screen for variation in repetitive DNA in the genome of *C. difficile*. We describe two tandem repeat loci, designated 'TR6' and 'TR10', which display extensive sequence variation that may be useful for sequence-based strain typing. Based on an investigation of 154 *C. difficile *isolates comprising 75 ribotypes, tandem repeat sequencing demonstrated excellent concordance with widely used PCR ribotyping and equal discriminatory power. Moreover, tandem repeat sequences enabled the reconstruction of the isolates' largely clonal population structure and evolutionary history.

**Conclusion:**

We conclude that sequence analysis of the two repetitive loci introduced here may be highly useful for routine typing of *C. difficile*. Tandem repeat sequence typing resolves phylogenetic diversity to a level equivalent to PCR ribotypes. DNA sequences may be stored in databases accessible over the internet, obviating the need for the exchange of reference strains.

## Background

*Clostridium difficile *is a Gram-positive, spore-forming, obligately anaerobic bacterium. It is the leading cause of nosocomial diarrhoea among patients undergoing antibiotic treatment [1,2]. The severity of *C. difficile*-associated disease (CDAD) ranges from mild diarrhoea to pseudomembranous colitis, toxic megacolon, and intestinal perforation [3-6]. Mortality rates of CDAD reportedly range from 6 to 30% [5,7,8]. During the last decade, the incidence of CDAD has increased significantly in North America [9-12] and Europe [4,8,13,14]. In the USA and Canada, this increase has been associated with the emergence of a novel, hypervirulent strain designated NAP1/027 [11,15]. Strains with the same genotype and associated outbreaks have also been reported from several European countries [14,16-18].

For infection control investigations and epidemiological studies, it is mandatory to track the emergence and spread of epidemic strains. For this purpose, appropriate genotyping methods are needed. The utility of a typing method will depend on its inter-laboratory reproducibility and data portability, its discriminatory power and concordance of identified groupings with epidemiology, the temporal stability of the genetic markers investigated, and the universal typeability of isolates [19]. Multilocus variable number of tandem repeats analysis (MLVA) is the most discriminatory method presently available for typing *C. difficile *[20,21]. Recently reported results suggested that the level of resolution achieved through MLVA may be highly useful for detecting epidemiological clusters of CDAD within and between hospitals [21,22]. The genetic loci currently exploited for MLVA-typing of *C. difficile *accumulate variation so rapidly, however, that longer-term relationships between isolates get obscured [23]. It is therefore advisable – and has been a common practice – to combine MLVA with the analysis of more conserved genetic markers [20-23]. Most commonly applied approaches to genotyping *C. difficile *at present are DNA macrorestriction analysis (based on pulsed-field gel electrophoresis, mostly used in Canada and the USA [12,15,24]) and PCR ribotyping (in Europe [25-27]). These two methods yield largely concordant results [23,27]. While DNA macrorestriction has slightly higher discriminatory power than PCR ribotyping, it is also more labour-intensive and time consuming [23,27-29].

A major disadvantage of PCR ribotyping, DNA macrorestriction, and other band-based typing techniques (including restriction endonuclease analysis (REA) [30]) is the poor portability and interlaboratory comparability of the generated data. Bacterial strains to be compared usually need to be run on the same electrophoresis gels, which requires the exchange of reference strains between institutions. This requirement seriously hampers epidemiological investigations, particularly at international scales [21,23].

Typing procedures based on DNA sequences overcome these limitations, since sequence data may easily be exchanged and stored in databases that are accessible via the internet. Accordingly, a scheme for multilocus sequence typing (MLST) of *C. difficile *was developed recently that is based on sequences from seven housekeeping gene fragments [31]. While MLST to date has been applied to a limited number of isolates, available data allowed a first glimpse at the largely clonal genetic population structure of *C. difficile *[23,31,32]. In clonal bacteria, novel genotypes in the course of evolution are generated primarily through mutations, which in slowly evolving housekeeping genes are rare. Hence, it is this very clonality of *C. difficile *and the associated linkage disequilibrium that causes MLST to provide poor discriminatory power, which is exemplified by the fact that relevant epidemic strains are not resolved [31]. In addition, MLST remains too expensive to be applied for routine typing aside from dedicated research projects.

More variable genomic regions may provide improved discrimination ability. In contrast to MLST, it may even suffice to sequence a single locus or very few genetic loci that are sufficiently variable, since – analysing a clonal population – phylogenetic inferences will rarely be confounded through homologous genetic recombination. Sequence-based typing schemes relying on one or several highly discriminatory markers have previously been established for a number of pathogens, including *Staphylococcus aureus *(*spa *gene) [33], *Campylobacter jejuni *(*flaA*) [34,35], *Streptococcus pyogenes *(*emm*) [36] and *Neisseria meningitidis *(*porA*, *fetA*) [37-39].

The surface layer protein gene *slpA *has recently been proposed as a promising target for sequence-based typing of *C. difficile *[40]. The limited data available suggests extremely high sequence variation among isolates and, correspondingly, excellent discriminatory power [23,40]. To date, however, *slpA *sequencing reportedly has been applied to a total of only 11 different ribotypes, and it is not clear if the method is universally applicable [23,40]. It is anticipated that the requirement for degenerate oligonucleotide primers may restrict the general utility of the current protocol [39]. The method has as yet not been successfully transferred to any other laboratory [23,40].

This present report describes the development and application of a new assay for genotyping *C. difficile *that is based on sequence analysis of two stretches of repetitive DNA. Investigating a panel of 154 diverse *C. difficile *isolates, we demonstrate extensive sequence variation in these genomic regions, resulting in high discriminatory power, and excellent concordance with PCR ribotyping.

## Results and discussion

### Identification of tandem repeat regions suitable for sequence-based typing

A total of 49 tandem repeat regions that met the selection criteria (repeat size, 15–40 bp; repeat copy number, > 5; consensus sequence match, < 90%) were detected in the genome sequence from strain 630 by using the program Tandem Repeats Finder version 4.00 [41,42]. For 36 of these repeat regions, it was possible to design PCR primers targeting flanking sequences, and from 28, PCR amplification products could reliably be generated from a panel of reference isolates. However, at 25 of these loci, sequence variation was insufficient to discriminate widely distributed strains, including ribotypes 027, 017, and 001 (not shown). The remaining three repeat regions could discriminate most of the ribotypes examined. The two most variable loci were designated TR6 and TR10 (Table 1). They are located at positions 0.7 Mb and 3.7 Mb of the *C. difficile *630 chromosome, respectively, and exhibited both, sequence and length polymorphisms. Locus TR6 is composed of 21-basepair repeat units and resides within an open reading frame encoding a hypothetical protein (orf CD0603 in the 630 genome sequence). A homology search in public databases did not identify any significant similarities with known proteins. In contrast, TR10 is located within a predicted non-coding region. It consists of 22-basepair repeats.

**Table 1:** Characteristics of tandem repeat loci TR6 and TR10.

tandem repeat locus	Location^a^	Size (bp)	Copy no. Range^b^	No. of different repeats^b^	Repeat consensus
TR6	725321 : 725600	21	7–37	80	CTTGCATACCACTAATAGTGC
TR10	3753166 : 3753574	22–23	4–26	51	AAATTAATTATTATATTTCTTT

^a ^Genome location based on *C. difficile *630 sequence http://www.sanger.ac.uk.

^b ^Based on analysis of 154 isolates typed in this study.

**Figure 1:**
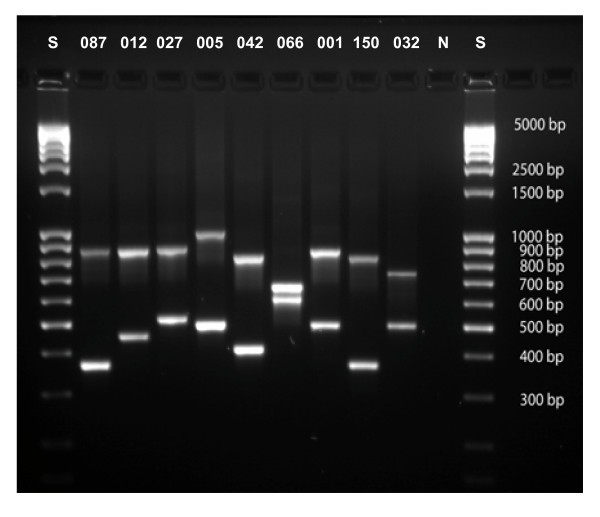
**Results from duplex PCR amplification of loci TR6 and TR10, performed on isolates representing various ribotypes as indicated**. **S**, 100 bp DNA ladder; **N**, negative control; **isolates (ribotypes)**: VPI10463 (087); 630 (012); NCTC 13366 (027); TR13 (005); N485 (042); SMI055 (066); NCTC 11204 (001); FR535 (150); FR505 (032).

**Figure 2:**
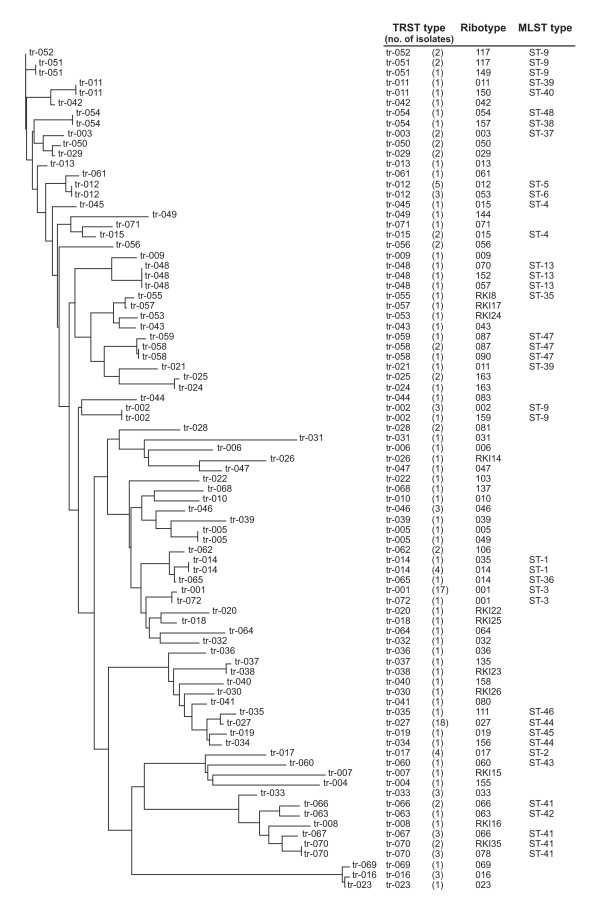
**Phylogenetic analysis (neighbor joining) based on the repeat successions in concatenated TR6 and TR10 sequences from 154 *C. difficile *isolates**. The repeat-distance matrix was calculated based on the DSI model, which considers repeat substitutions, insertions, deletions, and duplications (see Methods; [47]). Corresponding ribotypes, TRST types, and MLST sequence types are indicated.

We developed a DNA based typing scheme for *C. difficile *based on the sequence variation of TR6 and TR10. To facilitate the application of the tandem repeat sequence typing (TRST) scheme, a duplex PCR was designed which allowed simultaneous amplification of both loci (Figure 1). Sequence data were generated from duplex PCR products using the same primers as for amplification. Nucleotide sequences from TR6 and TR10 were concatenated and unique repeat successions were assigned distinct TRST types (tagged with consecutive numbers, prefixed with "tr"; Figure 2, Additional files 1, 2). A detailed comparison of TRST with PCR ribotyping is described in the following.

### Clonal evolution of tandem repeat regions

Genomic regions with short tandem repeat regions may evolve fast due to intra-molecular recombination and frequent polymerase slippage during DNA replication [43-45]. Accordingly, loci TR6 and TR10 displayed both, sequence polymorphisms, generated through exchange of individual nucleobases (Additional files 3, 4), and length polymorphisms, as a consequence of repeat copy number variation (Additional file 2). Sequences of individual repeats were highly variable, with a nucleotide diversity π of 0.28 ± 0.01 for TR6 and 0.23 ± 0.01 for TR10. The majority of nucleotide substitutions at locus TR6 were synonymous, i. e., they left the encoded amino acid sequence unaffected, and hence may be considered selectively neutral. This was reflected by a *Ka/Ks *value of 0.39, suggesting TR6 sequences evolve under purifying selection. Locus TR10 does not encode any protein and, hence, sequence variation likely is neutral, too.

**Figure 3:**
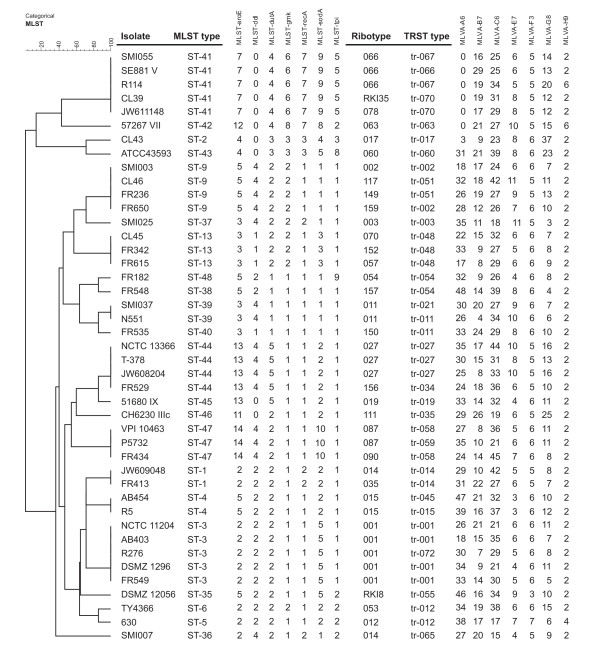
**Comparison of MLST, PCR ribotyping, TRST and MLVA for 43 *C. difficile *isolates**. Dendrogram is based on UPGMA analysis of MLST allelic profiles.

Furthermore, there is evidence of rare recombination between chromosomes from different strains, affecting tandem repeat sequences. One homologous recombination event apparently generated TRST type tr-021. While tr-021 shares an identical TR6 sequence with tr-011 (Additional file 2), its TR10 allele differs profoundly from that of tr-011 in both, length and sequence (Additional files 4 and 2), even though isolates displaying tr-011 (isolate N551) and tr-021 (SMI037) are affiliated to the same MLST type (ST-39) and ribotype (011; Figure 3). Interestingly, the TR10 allele of tr-021 is identical to the one of tr-005 (Additional file 2). Hence, the drastic difference between central parts of TR10 in tr-011 and tr-021 may be explained through a single event of horizontal gene transfer from an unrelated strain. Very similarly, tr-066 and tr-045 share identical alleles with closely related TRST types at either TR6 or TR10, respectively, yet differ drastically along a contiguous stretch of central repeats at the other tandem repeat locus. Again, identical alleles may be found elsewhere in the database (Additional file 2), suggesting they were horizontally transferred. In our dataset, these three TRST types displayed the only such discrepancies. We conclude that genetic recombination between unrelated chromosomes was involved in the evolution of maximally three TRST types out of 72 that were included in our set of isolates. Hence, the evolution of tandem repeats TR6 and TR10 is driven largely through clonal diversification, whereas the impact of recombination is extremely small. These results fully corroborate a previous estimate of a very low recombination rate in *C. difficile*, which had been based on MLST data [31].

While TR6 and TR10 displayed remarkable sequence variation, both loci seemed sufficiently stable to identify genetically related isolates collected over time. For one, the stability of TR6 and TR10 was demonstrated by two VPI 10463 and three 630 strains (including the published genome sequence), that prior to our analysis each had been handled in different laboratories (Additional file 1) and, hence, had independently been subcultured multiple times, but yet shared the same respective TRST sequence types (Additional file 1). Furthermore, stability of both tandem repeat regions was circumstantially suggested through identical sequences found in multiple isolates sharing the same ribotype but originating from different geographical regions (Additional file 1).

### Typeability, discriminatory power, and concordance with PCR ribotyping

Results were compared to PCR ribotyping on the basis of 154 isolates including international reference strains and clinical isolates collected at various German laboratories (Additional file 1). These isolates had been preselected from the material available to represent maximal diversity as judged on the basis of PCR ribotyping and geographic origin. They represented 75 different ribotypes (Additional file 1). Figure 2 shows a neighbor joining dendrogram based on the repeat successions in concatenated TR6 and TR10 sequences.

All 154 isolates were typeable by TRST. Considering both, differences in length and nucleotide sequence, 43 distinct alleles were identified at locus TR6, and 53 alleles at locus TR10 (Table 2, Additional file 2). Sequencing either one of the two loci had less discriminatory power than PCR ribotyping, as reflected by slightly lower discriminatory indices (0.93 and 0.95, respectively, versus 0.97 for ribotyping; Table 2). When considered in combination, however, sequence analysis of TR6 and TR10 resulted in the identification of 72 different TRST sequence types among the 154 isolates investigated (Additional file 2, Figure 2). This way, TRST and PCR ribotyping had equal discriminatory power, reflected by identical discriminatory indices (Table 2) based on the set of isolates included. It has to be considered, however, that this estimate will be skewed to some extent in favour of ribotyping, since ribotype diversity was the basis of initial isolate selection. Many ribotypes were represented by single isolates, and the potential ability of TRST to further discriminate within these ribotypes was thus not tested.

**Table 2:** Discriminatory power and concordance of tandem repeat sequence typing and PCR ribotyping.

Method	No. of strains included	No. of different types	Discriminatory index	95% CI	Concordance with ribotyping^a ^(%)
PCR ribotyping	154	75	0.967	0.953 – 0.982	n. a.
TRST^b^	154	72	0.967	0.954 – 0.981	89.8
TR6 sequencing	154	43	0.931	0.911 – 0.951	60.4
TR10 sequencing	154	53	0.949	0.934 – 0.964	71.6

^a ^Adjusted Rand's coefficient

^b ^Combination of sequences from TR6 and TR10.

TRST demonstrated high overall concordance with PCR ribotyping for the set of strains typed in this study, resulting in a calculated Adjusted Rand's index of 89.8% (Table 2). The probability that a pair of isolates with the same ribotype also shared identical TRST sequence types was 89.6% (Wallace index 0.896). Accordingly, ribotypes usually corresponded to specific TRST sequence types (Figure 2). For example, 18 isolates with ribotype 027, originating from six different European countries, displayed identical sequences at TR6 and TR10 that discriminated them from all other isolates, and jointly were assigned TRST sequence type tr-027 (Additional file 1, Figure 2). Similarly, four isolates with ribotype 017 from three different countries, including the reference strain for toxinotype VIII, were assigned sequence type tr-017 (Additional file 1, Figure 2). Future work on larger numbers of isolates may reveal that sequencing a single locus (TR6 or TR10) will suffice to identify epidemiologically relevant strains. For the sake of concordance with PCR ribotyping, however, we presently suggest to sequence both loci. As outlined above, this strategy will also detect the impact of recombination.

### Tandem repeat sequences are phylogenetically informative

Discrepancies between TRST and ribotyping were apparent where either method split a particular group of isolates into two or three classes, whereas the other lumped them into one (Figure 2). In virtually all of these cases, however, the respective isolates were affiliated to identical MLST sequence types or to single locus variants with respect to MLST (i. e., identical sequences at six out of seven MLST loci), indicating their close phylogenetic relatedness. Phylogenetic coherence of these additional (sub-)classes will remain unclear as long as there are no phylogenetic markers available to investigate the detailed evolutionary history of *C. difficile *within MLST sequence types.

**Figure 4:**
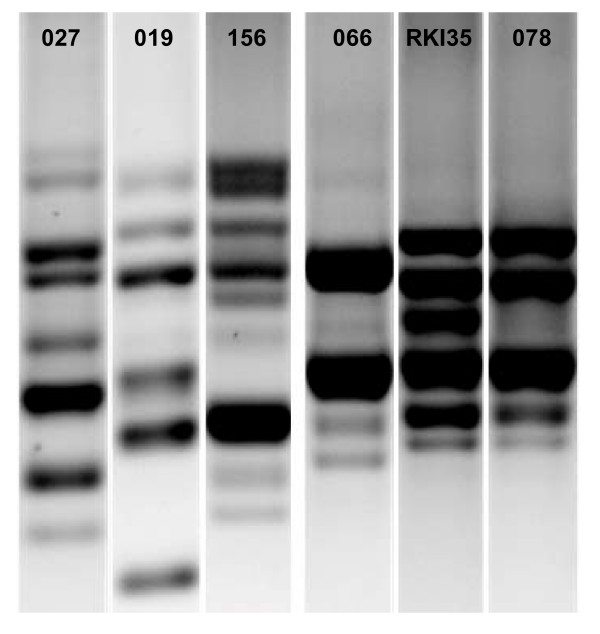
**PCR ribotyping band patterns of ribotypes 027 (isolate, NCTC 13366), 019 (51680), 156 (FR529), 066 (SE881), RKI35 (CL39) and 078 (JW611148)**.

MLVA typically resolves dozens of distinct genotypes within individual ribotypes [20,21]. However, MLVA provided little insight to the genetic relatedness within our collection, since almost all isolates differed from each other at four or more loci [20], even when they were affiliated to identical TRST sequence types or ribotypes (Figure 3). The sole useful exception was represented by isolates JW611148 and CL39, which shared identical alleles at five MLVA loci (Figure 3). The summed tandem-repeat difference between these two isolates was four repeats, which is below the threshold (= 10) previously suggested to indicate close genetic relationship based on MLVA [21]. MLST identity confirmed the relatedness of these isolates (Figure 3), and their close phylogenetic relationship also was correctly reflected by identical sequences at TR6 and TR10 (tr-070, Figure 3). However, these isolates displayed a distinct one-band difference between their ribotyping patterns, corresponding to ribotypes 078 and RKI35, respectively (Figure 4). This result illustrates the fact that ribotypes may differ widely with respect to the phylogenetic divergence they encompass. It may be noted that two other pairs of isolates shared highly similar MLVA patterns (AB403/CL45, NCTC11204/P5732; Figure 3). The summed tandem-repeat difference for the former pair is seven repeats, and hence these two isolates would be suggested to be extremely closely related based on MLVA alone [21]. These similarities, however, clearly reflect homoplasies, since MLST indicated these isolates were entirely unrelated (Figure 3). Thus, the application of MLVA as currently used is inappropriate when attempting to resolve distant phylogenetic relationships of *C. difficile *isolates. Again, in these cases, phylogeny was correctly indicated by TRST. We therefore conclude that it may be useful to combine TRST and MLVA in a nested hierarchical fashion, where TRST may resolve phylogenetic diversity to a level equivalent to PCR ribotypes, and MLVA may add additional resolution where desired.

Evolutionary relationships between isolates may be revealed through tandem repeat sequence alignment and phylogenetic analysis. This is also feasible for those isolates that were assigned different TRST types. For example, ribotypes 027, 156, and 019 by MLST are indicated to be closely related, since corresponding isolates are assigned two MLST sequence types that differ at one locus only (Figure 3). Close relationship of ribotypes 027 and 019 previously has also been found on the basis of DNA macrorestriction analysis, when isolates with both ribotypes were assigned to the 'North American Pulsotype NAP1' [23]. Concordantly with MLST and macrorestriction, TRST also indicated the relatedness of these types through similar tandem repeat sequences that clustered tightly in the phylogenetic tree (Figure 2), yet it maintained the discriminatory power of PCR ribotyping by assigning three different sequence types (tr-034, tr-027, tr-019) (Figure 2). Similarly, ribotypes 078 and RKI35 were indicated to be closely related to ribotype 066 by both, MLST and TRST (Figures 2 and 3). In contrast, these relationships were not at all apparent on the basis of ribotyping band patterns (Figure 4).

Phylogenetic relatedness was also indicated in cases where TRST was more discriminatory than PCR ribotyping. For example, ribotypes 001, 163, 087, 014, and 117 each were subdivided into several TRST types (Figure 2). Clusters of related tandem repeat sequences in the phylogenetic tree still corresponded to PCR ribotypes (Figure 2), which warrants the comparability of results from both methods. This feature may be highly desirable, since it will facilitate, for example, cross-referencing to ribotyping-based examinations and maintaining the continuity of ongoing surveillance programs.

Ribotyping does not enable phylogenetic analyses based on dissimilar banding patterns, and the relatedness of different ribotypes has not commonly been assessed. In the long run, large-scale mutation discovery and genomic (re-)sequencing will reveal the phylogenetic validity of typing procedures [46].

### Future prospects

We anticipate that PCR ribotyping will eventually be replaced by typing procedure(s) based on DNA sequences. The inherent portability of sequence data will obviate the need for the exchange of reference strains and enable decentralised genotyping efforts, which may boost large scale investigations on the molecular diversity of *C. difficile*. At present, however, our knowledge about the diversity and population biology of this important pathogen is very limited [23,31,32]. As a consequence, it is generally not clear if isolate groupings provided by various typing methods, including PCR ribotyping, are concordant with the epidemiology of associated disease [21,23]. Related to these considerations, one limitation of this present study is the lack of epidemiologically linked isolates in our data set. Investigations in the near future should evaluate the utility of tandem repeat sequencing for infection chain tracking and short-term epidemiological investigations.

## Conclusion

Sequence analysis of tandem repeats TR6 and TR10 provided full typeability across a wide range of *C. difficile *isolate diversity, excellent concordance with PCR ribotyping, and equal discriminatory ability. Sequence clades corresponded to phylogenetically coherent groupings. This sequencing-based typing approach may prove particularly useful because DNA sequences can easily be exchanged via the internet.

## Methods

### Bacterial isolates

A total of 154 *C. difficile *isolates comprising 75 different ribotypes were used in this study. The strain collection included both, international reference strains and selected clinical isolates from various German hospitals, collected in 2007 and 2008. More detailed information about individual isolates is given in Additional file 1.

### DNA extraction

Genomic DNA was isolated from cultures grown for 48 h on cycloserine-cefoxitin fructose agar (OXOID, Basingstoke, UK), by using the DNeasy Blood & Tissue Kit (QIAGEN, Hilden, Germany) according to the manufacturer's recommendations.

### PCR ribotyping

PCR ribotyping initially was performed at the Reference Laboratory for *Clostridium difficile *at the Leiden University Medical Center in the Netherlands and later was transferred to the Robert Koch Institute. We followed the protocol of Bidet et al. [26], except that PCR Products were run on 1.5% agarose gels in 1× TBE at 85 volts for 4 hours. Isolates were assigned novel PCR ribotypes if their patterns differed from previously named patterns by at least one band.

### Tandem repeat sequence typing (TRST)

To facilitate the application of tandem repeat sequence typing, a duplex PCR was designed using the following primers: TR6-F (5'-TTTCAACTTGTCCAGTTTTTAAGTC-3') and TR6-R (5'-ATGACATAGCGTTTGTGGAAT-3'); TR10-F (5'-TGCATCAAATTGGTCAAGACTC-3') and TR10-R (5'-TGAAATCATTGACTATAAAGCAAAA-3'). DNA amplification was performed on 1 μl of purified genomic DNA in a final volume of 50 μl containing 0.1 μM of TR6 and 1 μM of TR10 primers, 200 μM of each deoxynucleoside triphosphate, 1× PeqLab PCR buffer Y (20 mM Tris-HCL, 16 mM (NH_4_)_2_SO_4_, 0.01% Tween 20, 2 mM MgCl_2_) and 1.25 units Hot Taq-DNA-Polymerase (PeqLab, Erlangen, Germany). After an initial denaturation of 96°C for 3 min, the protocol consisted of 35 cycles at 96°C for 45 s, 52°C for 45 s, and 72°C for 45 s following a final extension at 72°C for 7 min. PCR products were prepared for sequencing using the QIAquick^® ^PCR Purification Kit (QIAGEN, Hilden, Germany) and 0.35 μl of the purified products were applied for sequencing using the BigDye Terminator v3.1 Cycle Sequencing Kit (Applied Biosystems, Foster City, USA) with identical primers employed in the PCR. Automated sequence detection was performed on an ABI capillary sequencing system and sequences were analysed using the BioNumerics 5.10 software (Applied Maths, Belgium).

### Classification of TRST types, repeat alignment, and cluster analysis

Data processing was performed with BioNumerics 5.10 by using a novel, dedicated "Repeat Typing" plugin that allowed automated batch assembly of trace files. The assignment of TRST sequence types was based on the successive occurrence of user-defined repeats in concatenated sequences from both tandem repeat loci. A repeat distance matrix for matching and clustering were calculated based on the DSI model [47], a mutation model comprising substitutions, indels (insertions or deletions), and duplications. Subsequent cluster analysis was performed based on the neighbor joining algorithm.

### Multilocus sequence typing

*Clostridium difficile *isolates were typed by MLST as described previously [31]. Sequence data were submitted to the *C. difficile *MLST database http://www.pasteur.fr/recherche/genopole/PF8/mlst/Cdifficile.html to assign allele profiles and the resulting sequence types. Sequence types were analysed by constructing a dendrogram based on the UPGMA (Unweighted Pair Group Method with Arithmetic mean) clustering algorithm using the multistate categorical similarity coefficient (tolerance 0%) available in the BioNumerics software.

### MLVA

Seven-locus MLVA was conducted as described previously [20,22], except that the different loci were PCR-amplified individually and PCR products were sequenced for repeat copy number determination. To facilitate sequence analysis of MLVA locus C6 [20], two novel oligonucleotide primers were used: C6-F 5'-CCAAGTCCCAGGATTATTGC-3' and C6-R 5'-AACATGGGGATTGGAATTGA-3'. Repeat copy numbers were determined manually using BioNumerics 5.10 software. The summed tandem-repeat difference was calculated where appropriate; it is the sum of repeat differences between two isolates at all seven MLVA loci [21].

### Discriminatory power, system concordance and molecular evolutionary analyses

An index of discrimination was calculated to compare the discriminating capacity of ribotyping, and TRST. The discriminatory index was defined as the average probability of two consecutively sampled strains being characterized as the same type. This probability depends on the number of strain types and their frequency distribution in the population. Discriminatory indices were calculated based on Simpson's index of diversity [48]. Confidence intervals for discriminatory indices were determined as described previously [49]. The Concordance of two typing schemes was calculated based on the adjusted Rand's and Wallace's coefficients [50]. While the Rand's coefficient allows a quantitative evaluation of the global congruence between two typing systems, the Wallace's coefficient compares the congruence of schemes depending on the directionality of typing by estimating the probability that a pair of isolates sharing the same type in system 1 also share the same type in system 2, and vice versa. Calculation of all parameters was performed with EpiCompare software, version 1.0 (Ridom GmbH, Würzburg, Germany).

The nucleotide diversity (π) and the ratio (*K*a/*K*s) of the average number of non-synonymous substitutions per non-synonymous site (*K*a) to the number to synonymous substitutions per synonymous site (*K*s) was calculated by using DnaSP, version 4.5 [51].

## Authors' contributions

HZ and UN designed research. HZ carried out the microbiological and molecular work. MR contributed reagents. DM and KJ devised analysis software. HZ, UN, EK, and CH performed data analyses. HZ, EK, MR, and UN wrote the manuscript.

## Supplementary Material

Additional File 1 **Bacterial isolates**. Table providing a list of bacterial isolates (isolate ID, source, geographic origin, PCR ribotype, TRST type, MLST type). Click here for file

Additional File 2 **TRST types and associated repeat profiles**. Table providing TRST types and associated repeat profiles. Click here for file

Additional File 3 **Locus TR6, individual repeat sequences identified from 154 isolates**. Table providing individual repeat sequences for locus TR6, identified from 154 isolates. Click here for file

Additional File 4 **Locus TR10, individual repeat sequences identified from 154 isolates**. Table providing individual repeat sequences for locus TR10, identified from 154 isolates. Click here for file

## References

[B1] Bartlett JG Antibiotic-associated pseudomembranous colitis Rev Infect Dis 1979 1 3 530 539 399365 10.1093/clinids/1.3.530

[B2] Thomas C Stevenson M Riley TV Antibiotics and hospital-acquired Clostridium difficile-associated diarrhoea: a systematic review J Antimicrob Chemother 2003 51 6 1339 1350 10.1093/jac/dkg254 12746372

[B3] Miller MA Hyland M Ofner-Agostini M Gourdeau M Ishak M Morbidity, mortality, and healthcare burden of nosocomial Clostridium difficile-associated diarrhea in Canadian hospitals Infect Control Hosp Epidemiol 2002 23 3 137 140 10.1086/502023 11918118

[B4] Kuijper EJ van Dissel JT Wilcox MH Clostridium difficile: changing epidemiology and new treatment options Curr Opin Infect Dis 2007 20 4 376 383 17609596 10.1097/QCO.0b013e32818be71d

[B5] Kyne L Hamel MB Polavaram R Kelly CP Health care costs and mortality associated with nosocomial diarrhea due to Clostridium difficile Clin Infect Dis 2002 34 3 346 353 10.1086/338260 11774082

[B6] Morgan OW Rodrigues B Elston T Verlander NQ Brown DF Brazier J Reacher M Clinical severity of Clostridium difficile PCR ribotype 027: a case-case study PLoS ONE 2008 3 3 e1812 18350149 10.1371/journal.pone.0001812 PMC2265541

[B7] Pepin J Valiquette L Cossette B Mortality attributable to nosocomial Clostridium difficile-associated disease during an epidemic caused by a hypervirulent strain in Quebec Cmaj 2005 173 9 1037 1042 16179431 10.1503/cmaj.050978 PMC1266326

[B8] Kuijper EJ Coignard B Tull P Emergence of Clostridium difficile-associated disease in North America and Europe Clin Microbiol Infect 2006 12 Suppl 6 2 18 10.1111/j.1469-0691.2006.01580.x 16965399

[B9] Zilberberg MD Shorr AF Kollef MH Increase in adult Clostridium difficile-related hospitalizations and case-fatality rate, United States, 2000–2005 Emerg Infect Dis 2008 14 6 929 931 18507904 10.3201/eid1406.071447 PMC2600276

[B10] McDonald LC Owings M Jernigan DB Clostridium difficile infection in patients discharged from US short-stay hospitals, 1996–2003 Emerg Infect Dis 2006 12 3 409 415 16704777 10.3201/eid1203.051064 PMC3291455

[B11] Loo VG Poirier L Miller MA Oughton M Libman MD Michaud S Bourgault AM Nguyen T Frenette C Kelly M A predominantly clonal multi-institutional outbreak of Clostridium difficile-associated diarrhea with high morbidity and mortality N Engl J Med 2005 353 23 2442 2449 10.1056/NEJMoa051639 16322602

[B12] Hubert B Loo VG Bourgault AM Poirier L Dascal A Fortin E Dionne M Lorange M A portrait of the geographic dissemination of the Clostridium difficile North American pulsed-field type 1 strain and the epidemiology of C. difficile-associated disease in Quebec Clin Infect Dis 2007 44 2 238 244 10.1086/510391 17173224

[B13] anonymous Deaths involving Clostridium difficle: England and Wales, 1999 and 2001–06 Health Stat Q 2008 37 52 56 18351026

[B14] Kuijper EJ Coignard B Brazier JS Suetens C Drudy D Wiuff C Pituch H Reichert P Schneider F Widmer AF Update of Clostridium difficile-associated disease due to PCR ribotype 027 in Europe Euro Surveill 2007 12 6 E1 2 17991399 10.2807/esm.12.06.00714-en

[B15] McDonald LC Killgore GE Thompson A Owens RC Jr Kazakova SV Sambol SP Johnson S Gerding DN An epidemic, toxin gene-variant strain of Clostridium difficile N Engl J Med 2005 353 23 2433 2441 10.1056/NEJMoa051590 16322603

[B16] Kuijper EJ Berg RJ van den Debast S Visser CE Veenendaal D Troelstra A Kooi T van der Hof S van den Notermans DW Clostridium difficile ribotype 027, toxinotype III, the Netherlands Emerg Infect Dis 2006 12 5 827 830 16704846 10.3201/eid1205.051350 PMC3374440

[B17] Barbut F Mastrantonio P Delmee M Brazier J Kuijper E Poxton I Prospective study of Clostridium difficile infections in Europe with phenotypic and genotypic characterisation of the isolates Clin Microbiol Infect 2007 13 11 1048 1057 10.1111/j.1469-0691.2007.01824.x 17850341

[B18] Zaiß NH Weile J Ackermann G Kuijper E Witte W Nübel U A case of Clostridium difficile-associated disease due to the highly virulent clone of Clostridium difficile PCR ribotype 027, March 2007 in Germany Euro Surveill 2007 12 11 E071115.1 18005641 10.2807/esw.12.46.03306-en

[B19] van Belkum A Tassios PT Dijkshoorn L Haeggman S Cookson B Fry NK Fussing V Green J Feil E Gerner-Smidt P Guidelines for the validation and application of typing methods for use in bacterial epidemiology Clin Microbiol Infect 2007 13 Suppl 3 1 46 10.1111/j.1469-0691.2007.01786.x 17716294

[B20] Berg RJ van den Schaap I Templeton KE Klaassen CH Kuijper EJ Typing and subtyping of Clostridium difficile isolates by using multiple-locus variable-number tandem-repeat analysis J Clin Microbiol 2007 45 3 1024 1028 17166961 10.1128/JCM.02023-06 PMC1829118

[B21] Marsh JW O'Leary MM Shutt KA Pasculle AW Johnson S Gerding DN Muto CA Harrison LH Multilocus variable-number tandem-repeat analysis for investigation of Clostridium difficile transmission in Hospitals J Clin Microbiol 2006 44 7 2558 2566 16825380 10.1128/JCM.02364-05 PMC1489528

[B22] Fawley WN Freeman J Smith C Harmanus C Berg RJ van den Kuijper EJ Wilcox MH Use of highly discriminatory fingerprinting to analyze clusters of Clostridium difficile infection cases due to epidemic ribotype 027 strains J Clin Microbiol 2008 46 3 954 960 18216211 10.1128/JCM.01764-07 PMC2268363

[B23] Killgore G Thompson A Johnson S Brazier J Kuijper E Pepin J Frost EH Savelkoul P Nicholson B Berg RJ van den Comparison of seven techniques for typing international epidemic strains of Clostridium difficile: restriction endonuclease analysis, pulsed-field gel electrophoresis, PCR-ribotyping, multilocus sequence typing, multilocus variable-number tandem-repeat analysis, amplified fragment length polymorphism, and surface layer protein A gene sequence typing J Clin Microbiol 2008 46 2 431 437 18039796 10.1128/JCM.01484-07 PMC2238077

[B24] Gal M Northey G Brazier JS A modified pulsed-field gel electrophoresis (PFGE) protocol for subtyping previously non-PFGE typeable isolates of Clostridium difficile polymerase chain reaction ribotype 001 J Hosp Infect 2005 61 3 231 236 10.1016/j.jhin.2005.01.017 16002184

[B25] Stubbs SL Brazier JS O'Neill GL Duerden BI PCR targeted to the 16S–23S rRNA gene intergenic spacer region of Clostridium difficile and construction of a library consisting of 116 different PCR ribotypes J Clin Microbiol 1999 37 2 461 463 9889244 10.1128/jcm.37.2.461-463.1999 PMC84342

[B26] Bidet P Barbut F Lalande V Burghoffer B Petit JC Development of a new PCR-ribotyping method for Clostridium difficile based on ribosomal RNA gene sequencing FEMS Microbiol Lett 1999 175 2 261 266 10.1111/j.1574-6968.1999.tb13629.x 10386377

[B27] Bidet P Lalande V Salauze B Burghoffer B Avesani V Delmee M Rossier A Barbut F Petit JC Comparison of PCR-ribotyping, arbitrarily primed PCR, and pulsed-field gel electrophoresis for typing Clostridium difficile J Clin Microbiol 2000 38 7 2484 2487 10878030 10.1128/jcm.38.7.2484-2487.2000 PMC86949

[B28] Spigaglia P Cardines R Rossi S Menozzi MG Mastrantonio P Molecular typing and long-term comparison of clostridium difficile strains by pulsed-field gel electrophoresis and PCR-ribotyping J Med Microbiol 2001 50 5 407 414 11339247 10.1099/0022-1317-50-5-407

[B29] Brazier JS The epidemiology and typing of Clostridium difficile J Antimicrob Chemother 1998 41 Suppl C 47 57 10.1093/jac/41.suppl_3.47 9630374

[B30] Clabots CR Johnson S Bettin KM Mathie PA Mulligan ME Schaberg DR Peterson LR Gerding DN Development of a rapid and efficient restriction endonuclease analysis typing system for Clostridium difficile and correlation with other typing systems J Clin Microbiol 1993 31 7 1870 1875 8394378 10.1128/jcm.31.7.1870-1875.1993 PMC265648

[B31] Lemee L Dhalluin A Pestel-Caron M Lemeland JF Pons JL Multilocus sequence typing analysis of human and animal Clostridium difficile isolates of various toxigenic types J Clin Microbiol 2004 42 6 2609 2617 15184441 10.1128/JCM.42.6.2609-2617.2004 PMC427854

[B32] Lemee L Bourgeois I Ruffin E Collignon A Lemeland JF Pons JL Multilocus sequence analysis and comparative evolution of virulence-associated genes and housekeeping genes of Clostridium difficile Microbiology 2005 151 Pt 10 3171 3180 10.1099/mic.0.28155-0 16207902

[B33] Shopsin B Gomez M Montgomery SO Smith DH Waddington M Dodge DE Bost DA Riehman M Naidich S Kreiswirth BN Evaluation of protein A gene polymorphic region DNA sequencing for typing of Staphylococcus aureus strains J Clin Microbiol 1999 37 11 3556 3563 10523551 10.1128/jcm.37.11.3556-3563.1999 PMC85690

[B34] Meinersmann RJ Helsel LO Fields PI Hiett KL Discrimination of Campylobacter jejuni isolates by fla gene sequencing J Clin Microbiol 1997 35 11 2810 2814 9350739 10.1128/jcm.35.11.2810-2814.1997 PMC230067

[B35] Price EP Thiruvenkataswamy V Mickan L Unicomb L Rios RE Huygens F Giffard PM Genotyping of Campylobacter jejuni using seven single-nucleotide polymorphisms in combination with flaA short variable region sequencing J Med Microbiol 2006 55 Pt 8 1061 1070 10.1099/jmm.0.46460-0 16849726

[B36] Beall B Facklam R Thompson T Sequencing emm-specific PCR products for routine and accurate typing of group A streptococci J Clin Microbiol 1996 34 4 953 958 8815115 10.1128/jcm.34.4.953-958.1996 PMC228924

[B37] Russell JE Jolley KA Feavers IM Maiden MC Suker J PorA variable regions of Neisseria meningitidis Emerg Infect Dis 2004 10 4 674 678 15200858 10.3201/eid1004.030247 PMC3323080

[B38] Thompson EA Feavers IM Maiden MC Antigenic diversity of meningococcal enterobactin receptor FetA, a vaccine component Microbiology 2003 149 Pt 7 1849 1858 10.1099/mic.0.26131-0 12855736

[B39] Elias J Harmsen D Claus H Hellenbrand W Frosch M Vogel U Spatiotemporal analysis of invasive meningococcal disease, Germany Emerg Infect Dis 2006 12 11 1689 1695 17283618 10.3201/eid1211.060682 PMC3372358

[B40] Kato H Yokoyama T Arakawa Y Typing by sequencing the slpA gene of Clostridium difficile strains causing multiple outbreaks in Japan J Med Microbiol 2005 54 Pt 2 167 171 10.1099/jmm.0.45807-0 15673512

[B41] Sebaihia M Wren BW Mullany P Fairweather NF Minton N Stabler R Thomson NR Roberts AP Cerdeno-Tarraga AM Wang H The multidrug-resistant human pathogen Clostridium difficile has a highly mobile, mosaic genome Nat Genet 2006 38 7 779 786 10.1038/ng1830 16804543

[B42] Benson G Tandem repeats finder: a program to analyze DNA sequences Nucleic Acids Res 1999 27 2 573 580 9862982 10.1093/nar/27.2.573 PMC148217

[B43] Levinson G Gutman GA Slipped-strand mispairing: a major mechanism for DNA sequence evolution Mol Biol Evol 1987 4 3 203 221 3328815 10.1093/oxfordjournals.molbev.a040442

[B44] Schlotterer C Evolutionary dynamics of microsatellite DNA Chromosoma 2000 109 6 365 371 10.1007/s004120000089 11072791

[B45] Eisen J Goldstein DB, Schötterer C Mechanistic basis for microsatellite instability Microsatellites: evolution and applications 1999 Oxford University Press, New York, NY 34 48

[B46] Nübel U Roumagnac P Feldkamp M Song J-H Ko KS Huang Y-C Coombs G Ip M Skov R Strommenger B Frequent emergence and limited geographic dispersal of methicillin-resistant *Staphylococcus aureus* Proc Nat Acad Sci USA 2008 105 14130 14135 10.1073/pnas.0804178105 18772392 PMC2544590

[B47] Benson G Sequence alignment with tandem duplication J Comput Biol 1997 4 3 351 367 9278065 10.1089/cmb.1997.4.351

[B48] Hunter PR Gaston MA Numerical index of the discriminatory ability of typing systems: an application of Simpson's index of diversity J Clin Microbiol 1988 26 11 2465 2466 3069867 10.1128/jcm.26.11.2465-2466.1988 PMC266921

[B49] Grundmann H Hori S Tanner G Determining confidence intervals when measuring genetic diversity and the discriminatory abilities of typing methods for microorganisms J Clin Microbiol 2001 39 11 4190 4192 11682558 10.1128/JCM.39.11.4190-4192.2001 PMC88515

[B50] Carrico JA Silva-Costa C Melo-Cristino J Pinto FR de Lencastre H Almeida JS Ramirez M Illustration of a common framework for relating multiple typing methods by application to macrolide-resistant Streptococcus pyogenes J Clin Microbiol 2006 44 7 2524 2532 16825375 10.1128/JCM.02536-05 PMC1489512

[B51] Nei M Gojobori T Simple methods for estimating the numbers of synonymous and nonsynonymous nucleotide substitutions Mol Biol Evol 1986 3 5 418 426 3444411 10.1093/oxfordjournals.molbev.a040410

