# Supplementary material for: Typing Clostridium difficile strains based on tandem repeat sequences
Source: BMC Microbiol. 2009 Jan 8;9:6. doi: 10.1186/1471-2180-9-6 (PMC2628660; doi:10.1186/1471-2180-9-6)
Supplement: Additional File 1 — Bacterial isolates. Table providing a list of bacterial isolates (isolate ID, source, geographic origin, PCR ribotype, TRST type, MLST type). [file 1471-2180-9-6-S1.pdf]

Additional file 1. Bacterial isolates.

Isolate	synonymous designation	source	geographic origin	PCR ribotype	TRST type	MLST type
07-00001	NCTC13366	NCTC	United Kingdom (Stoke Mandeville)	027	tr-027	ST-44
07-00002	NCTC13404	NCTC	United Kingdom	106	tr-062	
07-00003	NCTC11204	NCTC	United Kingdom (Sheffield)	001	tr-001	ST-3
07-00004	NCTC11205	NCTC	United Kingdom (Sheffield)	001	tr-001	ST-3
07-00005	NCTC11207	NCTC	United Kingdom (Sheffield)	001	tr-001	ST-3
07-00006	NCTC11223	NCTC	United Kingdom (Birmingham)	012	tr-012	ST-5
07-00007	NCTC12726	NCTC	United Kingdom (London)	039	tr-039	
07-00008	NCTC13287	NCTC	United Kingdom (Carlisle)	017	tr-017	
07-00009	NCTC13307 (630)	NCTC	Switzerland (Zürich)	012	tr-012	
07-00010	SMI002	T. Akerlund, Solna, Sweden	Sweden	012	tr-012	
07-00011	SMI003	T. Akerlund, Solna, Sweden	Sweden	002	tr-002	ST-9
07-00012	SMI007	T. Akerlund, Solna, Sweden	Sweden	014	tr-065	ST-36
07-00013	SMI011	T. Akerlund, Solna, Sweden	Sweden	117	tr-052	
07-00014	SMI012	T. Akerlund, Solna, Sweden	Sweden	010	tr-010	
07-00015	SMI014	T. Akerlund, Solna, Sweden	Sweden	001	tr-001	
07-00017	SMI025	T. Akerlund, Solna, Sweden	Sweden	003	tr-003	ST-37
07-00018	SMI035	T. Akerlund, Solna, Sweden	Sweden	014	tr-014	
07-00019	SMI037	T. Akerlund, Solna, Sweden	Sweden	011	tr-021	ST-39
07-00020	SMI044	T. Akerlund, Solna, Sweden	Sweden	015	tr-015	
07-00021	SMI046	T. Akerlund, Solna, Sweden	Sweden	016	tr-016	
07-00022	SMI047	T. Akerlund, Solna, Sweden	Sweden	029	tr-029	
07-00023	SMI055	T. Akerlund, Solna, Sweden	Sweden	066	tr-067	ST-41
07-00024	SMI073	T. Akerlund, Solna, Sweden	Sweden	081	tr-028	
07-00025	T-378	T. Akerlund, Solna, Sweden	Sweden (Huddinge)	027	tr-027	
07-00026	Ö99-1670	T. Akerlund, Solna, Sweden	Sweden (Örebro)	027	tr-027	
07-00027	A177:1	T. Akerlund, Solna, Sweden	Sweden (Malmö)	027	tr-027	ST-44
07-00028	TY4017	N. Nischik, Düsseldorf, Germany	Germany (Düsseldorf)	001	tr-001	
07-00031	TY4366	N. Nischik, Düsseldorf, Germany	Germany (Düsseldorf)	053	tr-012	ST-6
07-00033	TY4560	N. Nischik, Düsseldorf, Germany	Germany (Düsseldorf)	053	tr-012	
07-00034	TY4844	N. Nischik, Düsseldorf, Germany	Germany (Düsseldorf)	001	tr-001	
07-00037	TY4406	N. Nischik, Düsseldorf, Germany	Germany (Düsseldorf)	001	tr-001	
07-00038	SE844 (IIIa)	M. Rupnik, Maribor, Slovenia	Brüssel Belgium	080	tr-041	
07-00039	R10278 (IIIb)	M. Rupnik, Maribor, Slovenia	United Kingdom	027	tr-027	
07-00040	CH6230 (IIIc)	M. Rupnik, Maribor, Slovenia	United States (Chicago)	111	tr-035	ST-46
07-00041	SE881 (V)	M. Rupnik, Maribor, Slovenia	Belgium (Brüssel)	066	tr-066	ST-41
07-00042	51377 (VI)	M. Rupnik, Maribor, Slovenia	Belgium (Brüssel)	066	tr-067	
07-00043	57267 (VII)	M. Rupnik, Maribor, Slovenia	Belgium (Brüssel)	063	tr-063	ST-42
07-00044	1470 (VIII)	M. Rupnik, Maribor, Slovenia	Belgium (Brüssel)	017	tr-017	
07-00045	51680 (IX)	M. Rupnik, Maribor, Slovenia	Belgium (Brüssel)	019	tr-019	ST-45
07-00046	8864 (X)	M. Rupnik, Maribor, Slovenia	Belgium (Brüssel)	036	tr-036	
07-00047	IS58 (XIa)	M. Rupnik, Maribor, Slovenia	United Kingdom	033	tr-033	
07-00048	630 (0)	M. Rupnik, Maribor, Slovenia	Switzerland (Zürich)	012	tr-012	
07-00050	AB403	A. Becker, Karlsruhe, Germany	Germany (Karlsruhe)	001	tr-001	ST-3
07-00051	AB424	A. Becker, Karlsruhe, Germany	Germany (Karlsruhe)	001	tr-001	ST-3
07-00052	AB429	A. Becker, Karlsruhe, Germany	Germany (Karlsruhe)	117	tr-052	
07-00054	AB451	A. Becker, Karlsruhe, Germany	Germany (Karlsruhe)	002	tr-002	
07-00055	AB453	A. Becker, Karlsruhe, Germany	Germany (Karlsruhe)	001	tr-001	ST-3
07-00056	AB454	A. Becker, Karlsruhe, Germany	Germany (Karlsruhe)	015	tr-045	ST-4
07-00057	R5	H. Rüssmann, München, Germany	Germany (München)	015	tr-015	ST-4
07-00058	R37	H. Rüssmann, München, Germany	Germany (München)	078	tr-070	ST-41
07-00059	R79	H. Rüssmann, München, Germany	Germany (München)	016	tr-016	
07-00060	R114	H. Rüssmann, München, Germany	Germany (München)	066	tr-067	ST-41
07-00061	R276	H. Rüssmann, München, Germany	Germany (München)	001	tr-072	ST-3

07-00062	CL1	B. Schulte, Tübingen, Germany	Germany (Tübingen)	001	tr-001	
07-00063	CL7	B. Schulte, Tübingen, Germany	Germany (Tübingen)	001	tr-001	
07-00064	CL34	B. Schulte, Tübingen, Germany	Germany (Tübingen)	002	tr-002	
07-00065	CL36	B. Schulte, Tübingen, Germany	Germany (Tübingen)	163	tr-025	
07-00066	CL39	B. Schulte, Tübingen, Germany	Germany (Tübingen)	RKI35	tr-070	ST-41
07-00068	CL42	B. Schulte, Tübingen, Germany	Germany (Tübingen)	014	tr-014	
07-00069	CL43	B. Schulte, Tübingen, Germany	Germany (Tübingen)	017	tr-017	ST-2
07-00070	CL45	B. Schulte, Tübingen, Germany	Germany (Tübingen)	070	tr-048	ST-13
07-00071	CL46	B. Schulte, Tübingen, Germany	Germany (Tübingen)	117	tr-051	ST-9
07-00075	JW609048	J. Weile, Stuttgart, Germany	Stuttgart Germany	014	tr-014	ST-1
07-00076	JW611148	J. Weile, Stuttgart, Germany	Stuttgart Germany	078	tr-070	ST-41
07-00079	JW458241	J. Weile, Stuttgart, Germany	Stuttgart Germany	117	tr-051	
07-00080	JW608204	J. Weile, Stuttgart, Germany	Stuttgart Germany	027	tr-027	
07-00083	ATCC43593	ATCC	Belgium (Brüssel)	060	tr-060	ST-43
07-00084	DSMZ1296	DSMZ	United Kingdom (Sheffield)	001	tr-001	ST-3
07-00085	DSMZ12056	DSMZ	France (Paris)	RKI8	tr-055	ST-l
08-00001	VPI 10463	G. Ackermann, Leipzig, Germany	United States (Blacksburg)	087	tr-058	ST-47
08-00077	P5432	B. Bornhofen, Trier, Germany	Germany (Trier)	027	tr-027	
08-00084	N485	B. Bornhofen, Trier, Germany	Germany (Trier)	042	tr-042	
08-00095	N500	B. Bornhofen, Trier, Germany	Germany (Trier)	017	tr-017	
08-00098	N551	B. Bornhofen, Trier, Germany	Germany (Trier)	011	tr-011	ST-39
08-00104	P5732	B. Bornhofen, Trier, Germany	Germany (Trier)	087	tr-059	ST-47
08-00106	P5735	B. Bornhofen, Trier, Germany	Germany (Trier)	001	tr-001	
08-00120	N711	B. Bornhofen, Trier, Germany	Germany (Trier)	003	tr-003	
08-00124	P6084	B. Bornhofen, Trier, Germany	Germany (Trier)	081	tr-028	
08-00136	P6599	B. Bornhofen, Trier, Germany	Germany (Trier)	078	tr-070	
08-00140	P7034	B. Bornhofen, Trier, Germany	Germany (Trier)	RKI35	tr-070	
08-00148	P7165	B. Bornhofen, Trier, Germany	Germany (Trier)	027	tr-027	
08-00155		MVZ Leverkusen, Germany	Germany (Mechernich)	027	tr-027	
08-00164		T. Rieger, Essen, Germany	Germany (Essen)	046	tr-046	
08-00201		T. Mertes, Koblenz, Germany	Germany (Saarlouis)	027	tr-027	
08-00211		J. Heesemann, München, Germany	Germany (München)	001	tr-001	
08-00233		M. Herrmann, Homburg/Saar, Germany	Germany (Homburg/Saar)	027	tr-027	
08-00240		R. Schwarz, Köln, Germany	Germany (Köln)	001	tr-001	
08-00245		S. Swidsinski, Berlin, Germany	Germany (Berlin)	046	tr-046	
08-00246		S. Swidsinski, Berlin, Germany	Germany (Berlin)	046	tr-046	
08-00267		G. Enders, Stuttgart, Germany	Germany (Stuttgart)	027	tr-027	
DNA-1	597B (XXIV)	M. Rupnik, Maribor, Slovenia	unknown	RKI14	tr-026	
DNA-2	8785 (XXII)I	M. Rupnik, Maribor, Slovenia	unknown	RKI22	tr-020	
DNA-3	CH6223 (XXI)	M. Rupnik, Maribor, Slovenia	unknown	RKI15	tr-007	
DNA-4	J9965 (XVII)	M. Rupnik, Maribor, Slovenia	unknown	RKI23	tr-038	
DNA-5	K095 (XVIII)	M. Rupnik, Maribor, Slovenia	unknown	014	tr-014	
DNA-6	TR13 (XIX)	M. Rupnik, Maribor, Slovenia	unknown	005	tr-005	
DNA-7	TR14 (XX)	M. Rupnik, Maribor, Slovenia	unknown	163	tr-024	
DNA-8	55767 (IV)	M. Rupnik, Maribor, Slovenia	unknown	016	tr-016	
DNA-9	7325 (XXV)	M. Rupnik, Maribor, Slovenia	unknown	027	tr-027	
DNA-10	7459 (XXVI)	M. Rupnik, Maribor, Slovenia	unknown	050	tr-050	
DNA-11	A15	M. Rupnik, Maribor, Slovenia	unknown	066	tr-066	
DNA-12	AC008 (II)	M. Rupnik, Maribor, Slovenia	unknown	103	tr-022	
DNA-13	EX623 (I)	M. Rupnik, Maribor, Slovenia	unknown	RKI24	tr-053	
DNA-14	IS25 (XII)	M. Rupnik, Maribor, Slovenia	unknown	056	tr-056	
DNA-15	KK2443/2006 (XXVII)	M. Rupnik, Maribor, Slovenia	unknown	RKI17	tr-057	
DNA-16	R10870 (XIV)	M. Rupnik, Maribor, Slovenia	unknown	RKI25	tr-018	
DNA-17	R11402 (XIb)	M. Rupnik, Maribor, Slovenia	unknown	033	tr-033	
DNA-18	R9385 (XV)	M. Rupnik, Maribor, Slovenia	United Kingdom	RKI26	tr-030	

DNA-19	SUC36 (XVI)	M. Rupnik, Maribor, Slovenia	unknown	RKI16	tr-008	
DNA-20	VPI10463 (0)	M. Rupnik, Maribor, Slovenia	United States (Blacksburg)	087	tr-058	
FR179		M. Kist, Freiburg, Germany	Germany (Freiburg)	013	tr-013	
FR182		M. Kist, Freiburg, Germany	Germany (Freiburg)	054	tr-054	ST-48
FR236		M. Kist, Freiburg, Germany	Germany (Freiburg)	149	tr-051	ST-9
FR255		M. Kist, Freiburg, Germany	Germany (Freiburg)	071	tr-071	
FR342		M. Kist, Freiburg, Germany	Germany (Freiburg)	152	tr-048	ST-13
FR356		M. Kist, Freiburg, Germany	Germany (Freiburg)	083	tr-044	
FR402		M. Kist, Freiburg, Germany	Germany (Freiburg)	053	tr-012	
FR403		M. Kist, Freiburg, Germany	Germany (Freiburg)	023	tr-023	
FR406		M. Kist, Freiburg, Germany	Germany (Freiburg)	155	tr-004	
FR409		M. Kist, Freiburg, Germany	Germany (Freiburg)	056	tr-056	
FR413		M. Kist, Freiburg, Germany	Germany (Freiburg)	035	tr-014	ST-1
FR434		M. Kist, Freiburg, Germany	Germany (Freiburg)	090	tr-058	ST-47
FR460		M. Kist, Freiburg, Germany	Germany (Freiburg)	009	tr-009	
FR469		M. Kist, Freiburg, Germany	Germany (Freiburg)	047	tr-047	
FR486		M. Kist, Freiburg, Germany	Germany (Freiburg)	029	tr-029	
FR495		M. Kist, Freiburg, Germany	Germany (Freiburg)	061	tr-061	
FR505		M. Kist, Freiburg, Germany	Germany (Freiburg)	032	tr-032	
FR513		M. Kist, Freiburg, Germany	Germany (Freiburg)	031	tr-031	
FR529		M. Kist, Freiburg, Germany	Germany (Freiburg)	156	tr-034	ST-35
FR535		M. Kist, Freiburg, Germany	Germany (Freiburg)	150	tr-011	ST-40
FR545		M. Kist, Freiburg, Germany	Germany (Freiburg)	043	tr-043	
FR546		M. Kist, Freiburg, Germany	Germany (Freiburg)	069	tr-069	
FR548		M. Kist, Freiburg, Germany	Germany (Freiburg)	157	tr-054	ST-38
FR549		M. Kist, Freiburg, Germany	Germany (Freiburg)	001	tr-001	ST-3
FR581		M. Kist, Freiburg, Germany	Germany (Freiburg)	033	tr-033	
FR602		M. Kist, Freiburg, Germany	Germany (Freiburg)	050	tr-050	
FR615		M. Kist, Freiburg, Germany	Germany (Freiburg)	057	tr-048	ST-13
FR623		M. Kist, Freiburg, Germany	Germany (Freiburg)	137	tr-068	
FR650		M. Kist, Freiburg, Germany	Germany (Freiburg)	159	tr-002	ST-9
FR659		M. Kist, Freiburg, Germany	Germany (Freiburg)	006	tr-006	
FR702		M. Kist, Freiburg, Germany	Germany (Freiburg)	135	tr-037	
FR729		M. Kist, Freiburg, Germany	Germany (Freiburg)	064	tr-064	
FR740		M. Kist, Freiburg, Germany	Germany (Freiburg)	163	tr-024	
FR751		M. Kist, Freiburg, Germany	Germany (Freiburg)	103	tr-022	
FR798		M. Kist, Freiburg, Germany	Germany (Freiburg)	158	tr-040	
FR846		M. Kist, Freiburg, Germany	Germany (Freiburg)	106	tr-062	
FR861		M. Kist, Freiburg, Germany	Germany (Freiburg)	144	tr-049	
FR913		M. Kist, Freiburg, Germany	Germany (Freiburg)	049	tr-005	
K1710		M. Kist, Freiburg, Germany	Switzerland (Basel)	027	tr-027	
K1910		M. Kist, Freiburg, Germany	Switzerland (Basel)	027	tr-027	
K2006		M. Kist, Freiburg, Germany	Switzerland (Basel)	027	tr-027	
K769		M. Kist, Freiburg, Germany	Switzerland (Basel)	027	tr-027	
NL027		M. Kist, Freiburg, Germany	The Netherlands	027	tr-027	
genome	630	genome sequence	Switzerland (Zürich)	012	tr-012	ST-5
