# Supplementary material for: Typing Clostridium difficile strains based on tandem repeat sequences
Source: BMC Microbiol. 2009 Jan 8;9:6. doi: 10.1186/1471-2180-9-6 (PMC2628660; doi:10.1186/1471-2180-9-6)
Supplement: Additional File 2 — TRST types and associated repeat profiles. Table providing TRST types and associated repeat profiles. [file 1471-2180-9-6-S2.pdf]

Additional file 2. TRST types and associated repeat profiles.

TRST type	TR6 repeat succession	TR10 repeat succession
tr-001	R044-R011-R051-R007-R023-R024-R052-R053-R004-R019-R018-R019-R018-R022-R035	N003-N002-N004-N005-N004-N006-N004-N006-N004-N007-N004-N008-N009-N010-N004-N005-N004-N011
tr-002	R044-R011-R051-R007-R020-R007-R015-R008-R009-R022-R035	N003-N002-N004-N008-N009-N005-N004-N014-N015-N011
tr-003	R044-R011-R051-R007-R020-R021-R030-R007-R015-R008-R009-R022-R035	N003-N008-N004-N005-N004-N008-N004-N008-N009-N005-N009-N005-N009-N010-N004-N005-N004-N011
tr-004	R007-R003-R019-R018-R019-R018-R019-R018-R019-R018-R077-R018-R019-R018-R008-R028-R078-R028-R038-R079-R022-R080	N016-N017-N018-N035-N004-N005-N004-N005-N004-N002-N004-N002-N004-N005-N029-N038-N037-N018-N011
tr-005	R044-R011-R051-R007-R023-R024-R052-R053-R004-R019-R018-R019-R018-R022-R035	N003-N002-N004-N005-N004-N006-N004-N008-N004-N008-N004-N008-N004-N008-N004-N006-N004-N005-N009-N005-N004-N014-N015-N011
tr-006	R044-R011-R051-R007-R047-R024-R015-R053-R018-R019-R007-R019-R048-R019-R018-R032-R049-R038-R062-R022-R035	N003-N002-N004-N008-N004-N007-N004-N008-N009-N005-N004-N014-N015-N011
tr-007	R007-R012-R019-R018-R019-R018-R019-R041-R019-R007-R031-R007-R008-R028-R026-R028-R037-R028-R037-R036-R038-R071-R022-R035	N016-N017-N018-N042-N033-N004-N005-N004-N036-N004-N038-N037-N043-N011
tr-008	R043-R014-R013-R054-R010-R013-R054-R029-R039-R025-R054-R007-R025-R054-R010-R013-R054-R027-R013-R054-R042	N027-N017-N004-N028-N029-N002-N030-N031-N011
tr-009	R044-R011-R051-R007-R016-R007-R015-R008-R009-R022-R035	N003-N002-N004-N005-N004-N006-N004-N002-N004-N005-N012-N006-N009-N033-N009-N005-N009-N010-N004-N014-N004-N011
tr-010	R044-R011-R055-R007-R023-R056-R015-R053-R011-R019-R018-R019-R018-R022-R035	N003-N002-N004-N005-N004-N006-N004-N002-N004-N005-N012-N006-N009-N014-N015-N011
tr-011	R044-R011-R051-R007-R015-R019-R009-R022-R035	N003-N008-N004-N005-N004-N006-N004-N008-N009-N005-N009-N010-N004-N005-N004-N011
tr-012	R044-R007-R020-R007-R020-R021-R030-R007-R015-R008-R009-R022-R035	N003-N002-N004-N005-N012-N006-N019-N008-N004-N008-N009-N005-N009-N010-N004-N005-N004-N011
tr-013	R044-R007-R020-R007-R020-R021-R030-R007-R015-R008-R009-R022-R035	N003-N008-N004-N005-N004-N006-N004-N008-N004-N008-N004-N008-N009-N005-N009-N010-N004-N005-N004-N011
tr-014	R044-R011-R051-R007-R023-R024-R052-R053-R004-R019-R018-R019-R018-R022	N003-N002-N004-N005-N012-N006-N019-N008-N004-N008-N009-N005-N009-N010-N004-N005-N004-N011
tr-015	R044-R007-R020-R007-R020-R021-R030-R007-R015-R008-R009-R022-R035	N003-N002-N004-N005-N012-N006-N019-N008-N004-N008-N004-N008-N009-N010-N004-N014-N015-N011
tr-016	R044-R007-R051-R021-R063-R004-R050-R007-R063-R004-R050-R033-R003-R019-R033-R003-R064-R033-R003-R064-R033-R003-R064-R007-R019-R065-R006-R033-R003-R066-R001-R032-R067-R068-R069-R034-R035	N003-N008-N004-N020-N021-N011-N011
tr-017	R007-R003-R019-R018-R070-R018-R019-R018-R008-R028-R026-R028-R026-R028-R038-R071-R022-R035	N016-N017-N018-N011

tr-018	R044-R011-R051-R007-R023-R024-R052-R053-R004-R019-R018-R019-R018-R022-R035	N003-N002-N004-N005-N004-N006-N004-N005-N004-N008-N009-N005-N004-N023-N041-N011
tr-019	R044-R011-R051-R028-R040-R018-R032-R007-R032-R028-R028-R038-R072-R062-R022-R035	N003-N005-N004-N006-N004-N026-N026-N008-N022-N008-N009-N005-N004-N023-N004-N023-N024-N011
tr-020	R044-R011-R051-R007-R023-R024-R052-R053-R004-R019-R018-R019-R018-R022-R035	N003-N005-N004-N006-N004-N026-N008-N022-N008-N009-N005-N004-N023-N004-N023-N024-N011
tr-021	R044-R011-R051-R007-R015-R019-R009-R022-R035	N003-N002-N004-N005-N004-N006-N004-N008-N004-N008-N004-N008-N004-N008-N004-N006-N004-N005-N009-N005-N004-N014-N015-N011
tr-022	R044-R011-R055-R007-R023-R056-R015-R053-R011-R019-R058-R019-R018-R022-R035	N003-N002-N004-N005-N012-N006-N048-N049-N009-N005-N009-N005-N009-N010-N004-N008-N004-N011
tr-023	R044-R007-R051-R021-R063-R004-R050-R007-R063-R004-R050-R033-R003-R019-R033-R003-R064-R033-R003-R064-R033-R003-R064-R007-R019-R065-R006-R033-R003-R066-R001-R032-R067-R068-R069-R034-R035	N003-N008-N004-N020-N021-N011
tr-024	R044-R011-R051-R004-R019-R018-R019-R018-R022-R035	N003-N002-N004-N005-N012-N006-N004-N008-N004-N008-N004-N006-N004-N005-N009-N032-N004-N005-N004-N011
tr-025	R044-R011-R051-R004-R019-R018-R019-R018-R022-R035	N003-N002-N004-N005-N012-N006-N004-N006-N004-N008-N004-N008-N004-N006-N004-N005-N009-N032-N004-N005-N004-N011
tr-026	R044-R011-R051-R007-R016-R011-R073-R024-R015-R053-R011-R051-R004-R019-R074-R019-R048-R019-R018-R032-R049-R038-R062-R022-R035	N003-N002-N004-N005-N004-N006-N004-N002-N004-N005-N012-N006-N009-N033-N009-N039-N009-N010-N004-N014-N004-N011
tr-027	R044-R011-R051-R028-R040-R018-R032-R007-R032-R028-R028-R038-R072-R062-R022-R035	N003-N002-N004-N005-N004-N006-N009-N026-N008-N022-N008-N009-N005-N004-N023-N004-N023-N024-N011
tr-028	R044-R011-R051-R007-R023-R024-R019-R048-R019-R018-R075-R049-R038-R062-R022-R035	N003-N008-N004-N005-N004-N006-N004-N008-N004-N008-N009-N005-N009-N005-N009-N010-N004-N014-N004-N011
tr-029	R044-R007-R020-R007-R020-R021-R030-R007-R015-R008-R009-R022-R035	N003-N008-N004-N005-N004-N008-N004-N008-N009-N005-N009-N005-N009-N010-N004-N014-N004-N011
tr-030	R044-R011-R051-R028-R040-R018-R032-R007-R076-R028-R038-R062-R022-R035	N003-N008-N004-N006-N004-N026-N008-N022-N008-N009-N005-N004-N023-N004-N023-N024-N011
tr-031	R044-R011-R051-R007-R016-R011-R055-R007-R016-R011-R051-R007-R045-R011-R055-R007-R023-R007-R015-R053-R051-R004-R019-R018-R019-R060-R019-R018-R032-R061-R038-R062-R022-R035	N003-N002-N004-N005-N012-N006-N019-N008-N004-N014-N015-N011
tr-032	R044-R011-R051-R059-R023-R024-R052-R053-R004-R019-R018-R019-R018-R022-R035	N003-N002-N004-N005-N004-N006-N004-N046-N004-N014-N045-N011
tr-033	R043-R014-R013-R054-R010-R013-R054-R029-R039-R025-R035	N027-N017-N004-N028-N029-N002-N030-N031-N011
tr-034	R044-R011-R051-R028-R040-R018-R032-R007-R032-R028-R028-R038-R072-R062-R022-R035	N003-N002-N004-N006-N004-N006-N004-N026-N008-N022-N008-N009-N005-N004-N023-N004-N023-(N004)-N011
tr-035	R044-R011-R051-R028-R040-R018-R032-R007-R032-R007-R032-R028-R038-R072-R062-R022-R035	N025-N002-N004-N005-N004-N006-N009-N026-N008-N022-N008-N009-N005-N004-N023-N004-N023-N024-N011

tr-036	R044-R011-R051-R028-R040-R018-R032-R028-R028-R028-R038-R062-R022-R035	N003-N002-N004-N005-N004-N006-N022-N008-N009-N026-N023-N004-N023-N024-N011
tr-037	R044-R011-R051-R028-R040-R018-R032-R007-R032-R007-R032-R028-R028-R038-R062-R022-R035	N003-N002-N004-N005-N004-N044-N004-N006-N022-N005-N004-N023-N004-N023-N004-N023-N024-N011
tr-038	R044-R011-R051-R028-R040-R018-R032-R007-R032-R007-R032-R028-R038-R062-R022-R035	N003-N002-N004-N005-N004-N044-N004-N006-N022-N005-N004-N023-N004-N023-N004-N023-N024-N011
tr-039	R044-R011-R055-R007-R023-R056-R015-R053-R011-R019-R018-R019-R018-R022-R035	N003-N002-N004-N005-N012-N013-N013-N006-N004-N006-N004-N008-N004-N008-N004-N008-N004-N006-N004-N005-N009-N005-N004-N014-N015-N011
tr-040	R044-R011-R051-R002-R040-R018-R032-R007-R032-R028-R005-R028-R036-R038-R062-R022-R035	N003-N005-N004-N006-N004-N005-N022-N008-N009-N005-N004-N023-N004-N023-N024-N011
tr-041	R044-R011-R051-R028-R040-R018-R032-R007-R032-R028-R038-R072-R062-R022-R035	N003-N005-N004-N006-N022-N008-N009-N005-N004-N023-N004-N023-N024-N011
tr-042	R044-R007-R020-R007-R015-R008-R009-R022-R035	N003-N008-N004-N005-N004-N006-N004-N008-N009-N005-N009-N010-N004-N005-N004-N011
tr-043	R044-R011-R051-R007-R015-R019-R009-R022-R035	N003-N002-N004-N005-N004-N006-N004-N002-N004-N008-N009-N005-N004-N008-N009-N005-N004-N014-N015-N011
tr-044	R044-R011-R051-R007-R023-R021-R030-R017-R015-R008-R009-R022-R035	N003-N008-N004-N005-N004-N011
tr-045	R044-R007-R020-R007-R020-R021-R030-R007-R015-R008-R009-R022-R035	N003-N002-N004-N005-N004-N006-N004-N006-N004-N007-N004-N008-N009-N010-N004-N005-N004-N011
tr-046	R044-R011-R051-R007-R023-R024-R052-R053-R004-R019-R018-R019-R018-R022-R035	N003-N002-N004-N005-N004-N006-N004-N002-N004-N008-N009-N005-N004-N008-N009-N005-N004-N014-N015-N011
tr-047	R044-R011-R051-R007-R023-R024-R015-R053-R004-R019-R018-R019-R048-R019-R018-R032-R049-R038-R062-R022-R035	N003-N002-N004-N005-N004-N006-N004-N002-N004-N005-N012-N006-N009-N005-N009-N005-N009-N010-N004-N014-N004-N011
tr-048	R044-R011-R051-R007-R020-R021-R030-R007-R015-R008-R009-R022-R035	N003-N002-N004-N005-N004-N006-N004-N002-N004-N005-N012-N006-N009-N033-N009-N005-N009-N010-N034-N014-N024-N011
tr-049	R044-R007-R020-R007-R020-R021-R030-R007-R015-R008-R009-R022-R035	N050-N002-N004-N008-N004-N051-N009-N051-N004-N006-N004-N008-N004-N046-N034-N014-N024-N011
tr-050	R044-R007-R020-R007-R020-R021-R030-R007-R015-R008-R009-R022-R035	N003-N040-N004-N005-N004-N008-N004-N008-N009-N005-N009-N005-N009-N010-N004-N014-N004-N011
tr-051	R044-R007-R020-R021-R030-R007-R015-R008-R009-R022-R035	N003-N008-N004-N005-N004-N006-N009-N008-N009-N005-N009-N010-N004-N005-N004-N011
tr-052	R044-R007-R020-R021-R030-R007-R015-R008-R009-R022-R035	N003-N008-N004-N005-N004-N006-N004-N008-N009-N005-N009-N010-N004-N005-N004-N011
tr-053	R044-R007-R015-R008-R009-R022-R035	N003-N002-N004-N005-N004-N006-N004-N002-N004-N008-N009-N005-N004-N008-N009-N005-N004-N014-N015-N011
tr-054	R044-R007-R020-R021-R030-R007-R015-R008-R009-R022-R035	N003-N008-N004-N007-N004-N008-N009-N010-N004-N014-N004-N011
tr-055	R044-R007-R020-R007-R020-R007-R015-R008-R009-R022-R035	N003-N002-N004-N005-N004-N006-N004-N002-N003-N008-N009-N005-N004-

		N008-N009-N005-N004-N014-N015-N011
tr-056	R044-R011-R051-R007-R020-R021-R030-R007-R015-R008-R009-R022-R035	N003-N002-N004-N005-N012-N006-N004-N006-N004-N008-N004-N008-N009-N006-N004-N006-N004-N008-N004-N011
tr-057	R044-R007-R020-R007-R020-R007-R015-R008-R009-R022-R035	N003-N002-N004-N005-N004-N006-N004-N002-N004-N008-N009-N005-N004-N008-N009-N005-N004-N014-N015-N011
tr-058	R044-R011-R051-R007-R015-R019-R009-R022-R035	N003-N002-N004-N008-N004-N006-N004-N006-N004-N007-N004-N008-N009-N005-N004-N014-N015-N011
tr-059	R044-R011-R051-R007-R015-R019-R046-R022-R035	N003-N002-N004-N008-N004-N006-N004-N006-N004-N007-N004-N008-N009-N005-N004-N014-N015-N011
tr-060	R007-R003-R008-R028-R026-R028-R008-R007-R008-R028-R026-R028-R026-R028-R038-R071-R022-R035	N016-N017-N018-N035-N004-N005-N004-N005-N004-N036-N004-(N002)-N038-N037-N018-N011
tr-061	R044-R007-R020-R007-R020-R021-R030-R007-R015-R008-R009-R022-R035	N003-N002-N004-N005-N012-N006-N019-N008-N004-N008-N009-N005-N009-N010-N004-N014-N045-N011
tr-062	R057-R011-R051-R007-R023-R024-R015-R053-R004-R019-R018-R019-R018-R022-R035	N003-N002-N004-N005-N012-N006-N019-N008-N004-N008-N009-N005-N009-N010-N004-N005-N004-N011
tr-063	R043-R014-R013-R054-R010-R013-R054-R042-R029-R039-R025-R054-R010-R013-R054-R042-R029-R039-R025-R035	N027-N017-N004-N028-N029-N002-N030-N031-N011
tr-064	R044-R011-R051-R007-R023-R024-R052-R053-R004-R019-R018-R019-R018-R022-R035	N047-N017-N018-N011
tr-065	R044-R011-R051-R007-R023-R024-R052-R053-R004-R019-R018-R019-R018-R022-R035	N003-N002-N004-N005-N012-N006-N019-N008-N004-N008-N009-N005-N009-N010-N004-N005-N004-N011
tr-066	R043-R014-R013-R054-R010-R013-R054-R042-R039-R025-R054-R042-R010-R013-R054-R042-R042-R039-R025-R035	N027-N017-N004-N028-N029-N002-N030-N031-N011
tr-067	R043-R014-R013-R054-R042-R029-R039-R025-R054-R027-R013-R054-R042-R010-R013-R054-R027-R013-R035	N027-N017-N004-N028-N029-N002-N030-N031-N011
tr-068	R044-R011-R055-R007-R023-R056-R015-R053-R011-R019-R018-R019-R018-R022-R035	N003-N008-N009-N005-N009-N005-N004-N008-N004-N008-N009-N006-N009-N005-N004-N014-N015-N011
tr-069	R044-R007-R051-R021-R063-R004-R050-R007-R063-R004-R050-R033-R003-R019-R033-R003-R064-R007-R003-R064-R033-R003-R064-R007-R019-R065-R006-R033-R003-R066-R001-R032-R067-R068-R069-R034-R035	N003-N008-N004-N020-N021-N011-N011
tr-070	R043-R014-R013-R054-R010-R013-R054-R042-R029-R039-R025-R054-R007-R025-R054-R042-R010-R013-R054-R027-R013-R035	N027-N017-N004-N028-N029-N002-N030-N031-N011
tr-071	R044-R007-R020-R007-R020-R021-R030-R007-R015-R008-R009-R022-R035	N003-N002-N004-N005-N012-N006-N019-N008-N004-N014-N015-N011
tr-072	R044-R011-R051-R007-R023-R024-R052-R053-R004-R019-R018-R019-R018-R022-R035	N003-N002-N004-N005-N004-N006-N004-N007-N004-N008-N009-N010-N004-N005-N004-N011
