# Supplementary material for: Typing Clostridium difficile strains based on tandem repeat sequences
Source: BMC Microbiol. 2009 Jan 8;9:6. doi: 10.1186/1471-2180-9-6 (PMC2628660; doi:10.1186/1471-2180-9-6)
Supplement: Additional File 3 — Locus TR6, individual repeat sequences identified from 154 isolates. Table providing individual repeat sequences for locus TR6, identified from 154 isolates. [file 1471-2180-9-6-S3.pdf]

Additional file 3. Locus TR6, individual repeat sequences identified from 154 isolates.

>R001	>R017	>R033	>R049	>R065
CTTGCATACCACTAATAGTGC	CTTGCATATCGCTAATAGTGA	CTTGCATATCGCTAATATTGC	CTTGCATATTGCTAATAGTAC	TTTGTATATCGCTAATAGTGC
>R002	>R018	>R034	>R050	>R066
CTTGCATATCACTAATAGTAC	TTTGCATATCGCTAATAGTGC	CTTCAATACCACTGATACTAC	CATCCATACCACTGATATTGC	TTTCAATACCATTGATATTGC
>R003	>R019	>R035	>R051	>R067
CTTGCATGCCACTAATAGTGC	CTTCGATACCATTGATATTGC	CTTGCATATTACTAATATTAC	CATCCATACTACTGATACTGC	CTTGCATATCGCTAATATTAC
>R004	>R020	>R036	>R052	>R068
CTTGCATACTACTAATAGTGC	CATTCATACTACTGATACTGC	CTTGCATATCGCCAATAGTAC	CTTGCATACCACTAATAGTGT	CTTGCATATCGTTAATATTAC
>R005	>R021	>R037	>R053	>R069
CCTGCATATCGCTAATAGTAC	CTTGCATATCGCTAATAGTGT	CTTCAATGCCATTGATACTGC	TTTCGATACCATTGATATTGC	CCTGCATGCCATTAACAGTGC
>R006	>R022	>R038	>R054	>R070
CTTCAATACCACTGATGCTGC	CTTCGATACCACTGATACTAC	CTTGCATATAGTTAATATTAC	CTTGCATATCGCCAATATTAC	TTTCGATACCATTGATATTAC
>R007	>R023	>R039	>R055	>R071
CTTGCATATCGCTAATAGTGC	CATTCATACCACTGATACTAC	CTTGCACGCTGCTAATAGTGC	CATCCATACTACTGATACTTC	CCTGCATGCCGTTAACAGTGC
>R008	>R024	>R040	>R056	>R072
CTTCGATACCATTAATATTGC	CTTGCATATCGCCAATAGTGC	CTTCGATATCATTGATATTGC	CTTGCATATCTCCAATAGTGC	CCTGCATATAGTTAATATTAC
>R009	>R025	>R041	>R057	>R073
TTTGCATATCACTAATAGTGC	CCTCTATACCACTGATACTGC	TTTGCATATCGCCAATAGTGC	CTTGCATATCAGTAATACTGC	CATCCATACTACTGATACTAC
>R010	>R026	>R042	>R058	>R074
CTTGCATGCCGCTAATAGTGC	CTTCAATACCATTGATACTGC	CTTGCATATCGCTGATAGTAC	TTTGAATATCGCTAATAGTGC	TTTGCATACTACTAATAGTGC
>R011	>R027	>R043	>R059	>R075
CTTGCATACTATTAATAGTGC	CTTGCATATCGCTGATAGTGC	CTTGCATATCACCAATATTAC	CTTGAATATCGCTAATAGTGC	CTTCGATACCATTGATACTAC
>R012	>R028	>R044	>R060	>R076
CTTGCATGCCACTAATATTGC	CTTGCATATCGCTAATAGTAC	CTTGCATATCAGTAATACTGT	TTTGCGTATCGCTAATAGTGT	CTTCGATACCATTTATACTGC
>R013	>R029	>R045	>R061	>R077
CTTCTATACCACTGATACTGC	CTTGCATATCGATGATAGTAC	CATCCATACCACTGATACTGC	CTTGCATATTGCTAATATTAC	CTTCGATACCATTGATATTAC
>R014	>R030	>R046	>R062	>R078
CTTGCATGCCACTAATAGTGA	CATCTATACCACTGATACTGC	TTTGCATATTACTAATAGTGC	CCTGCATGCCATTAACAGTAC	CTTCAATAACATTGATACTGC
>R015	>R031	>R047	>R063	>R079
CTTGCATGCCACTAATAGTGT	TTTCGATACCATTAATATTGC	CATCTATACCACTGATACTAC	CATCCATGCCACTGATACTGC	CCTGAATGCCATTAACAGTGC
>R016	>R032	>R048	>R064	>R080
CATTCATACCACTGATACTGC	CTTCGATACCATTGATACTGC	TTTGCATATCGCTAATAGTGT	CTTCGATACCACTGATACTGC	CTTGCATATTACTAATGTTAC
