# Supplementary material for: Typing Clostridium difficile strains based on tandem repeat sequences
Source: BMC Microbiol. 2009 Jan 8;9:6. doi: 10.1186/1471-2180-9-6 (PMC2628660; doi:10.1186/1471-2180-9-6)
Supplement: Additional File 4 — Locus TR10, individual repeat sequences identified from 154 isolates. Table providing individual repeat sequences for locus TR10, identified from 154 isolates. [file 1471-2180-9-6-S4.pdf]

Additional file 4. Locus TR10, individual repeat sequences identified from 154 isolates.

>N001	>N017	>N033	>N049
AAATTAATTATTATATTTCTTT	AAATTAATTTTCTATGTTTCTT	AGATTAGTTTTCTATACTTCCT	AAATTAATTTTCTATCCTTCCT
>N002	>N018	>N034	>N050
AAATTAATTTTCTATATTTCTT	AAATTAGCTTATTATACTTTTT	AAATTAGTTTATTATGCTTCTTT	AAATTAATGTATTGTGTTTCTTT
>N003	>N019	>N035	>N051
AAATTAATGTATTGTATTTCTTT	GAATTAGTTTATTATACTTCTTT	AAATTAATTTTTTATACTTCCT	AGATTAATTTCCTATACTTCCT
>N004	>N020	>N036	
AAATTAGTTTATTATACTTCTTT	AGATTAATTTTCTATATTTCTT	AGATTAATTTTCCATACTTCCT	
>N005	>N021	>N037	
AGATTAATTTTCTATACTTCCT	AAATTAGTTTATTATACTTCCT	AAATTAGCTCATTATACTTCTTT	
>N006	>N022	>N038	
AGATTAGCTTTCTATACTTCCT	AAATTAGTTCATTATACTTCTTT	AAATTAATTTTCCATATTTCTT	
>N007	>N023	>N039	
AGATTAGCTTTCTATATTTCTT	AAATTAATTTTCTACACTTCCT	AGATTAATTTTCTATCCTTCCT	
>N008	>N024	>N040	
AAATTAATTTTCTATACTTCCT	AAATTAGTTTATTATATTTCTT	AAACTAATTTTCTATACTTCCT	
>N009	>N025	>N041	
AAATTAGTTTATTACACTTCTTT	AAATTAATATATTGTATTTCTTT	AAATTAGTTTATTATACTTTTT	
>N010	>N026	>N042	
AGATTAATTTTCTATACTTTCT	AGATTAATTTTCTATACTTCTTT	AAATTAATTTTTTATACTTCTTT	
>N011	>N027	>N043	
AAATTAATTTTCTATACTTCTT	AAGTTAATTTATTGTATTTCTTT	AAATTAGCTTATTATATTTCTT	
>N012	>N028	>N044	
AAATTAGTTTACTATACTTCTTT	AGATTAATTTTCTATATTTCCT	AGATTAGCTTTCTATACTTTCT	
>N013	>N029	>N045	
AGATTAGCTTTCTATACTTCTTT	AAATTAATTTATTATATTTCTTT	AAATTAGTTTATTATACTTATTT	
>N014	>N030	>N046	
AGATTAATTTTCTACACTTCCT	AAGTTAGCTCATTATACTTCTTT	AAATTAATTTTCTATACTTTCT	
>N015	>N031	>N047	
AAATTAATTTATTATATTTTTT	AAATTAGCTTATTATACTTCTT	AAATTAATTATTGTGTTTCTTT	
>N016	>N032	>N048	
AAATTAATTTATTGTATTTCTTT	AGATTAACTTTCTATACTTTCT	GAATTAGTTTATTATACTCCTTT	
